# Aminopeptidase N: a multifunctional and promising target in medicinal chemistry

**DOI:** 10.1039/d5ra03038b

**Published:** 2025-07-23

**Authors:** Oldřich Farsa, Tomáš Uher

**Affiliations:** a Department of Chemical Drugs, Faculty of Pharmacy, Masaryk University Palackého 1946/1 Brno Czech Republic farsao@pharm.muni.cz

## Abstract

Aminopeptidase N (APN) is a zinc metalloproteinase present in almost all types of organisms and has various functions. Many of them are unrelated to its catalytic activity, which is why APN is sometimes classified as a moonlighting enzyme. APN is involved in carcinogenesis and angiogenesis. It also works as an entrance receptor for some coronaviruses and acts as a mediator during skin inflammation. Additionally, APN removes and helps to recycle regulatory proteins including neuropeptides, which is why its inhibitors hold therapeutic potential for a wide range of diseases, making the design and development of such molecules highly desirable. Some of them, such as bestatin or tosedostat, have already been tested as therapeutics with partial success. This article aims to bring an overview of multiple APN functions and implications for various diseases and their inhibitors which have already been prepared, and to suggest areas where the development of inhibitors may be promising in the future.

## Abstract 2

Aminopeptidase N (APN) is a zinc metalloproteinase present in almost all types of organisms and has various functions.

## Graphical abstract

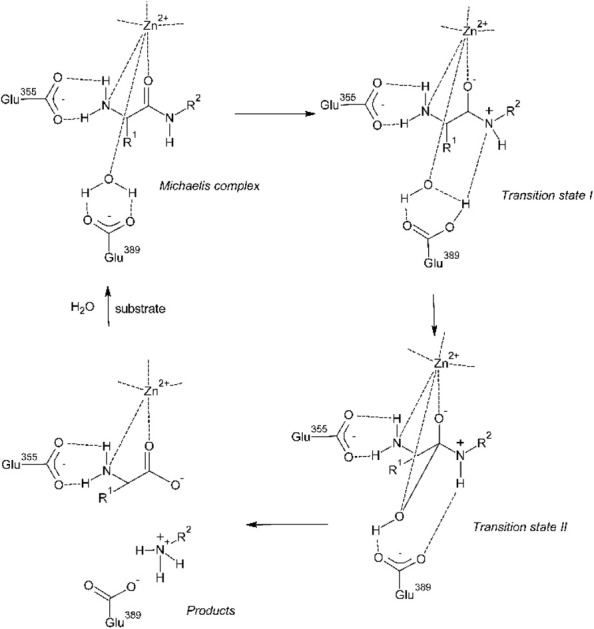

## A. Introduction

### Discovery and history of APN

The first reports of APN^1^ were made by Binkley and colleagues in 1952 as peptidase activity was present in seemingly purified RNA preparations.^2^ The same team isolated the enzyme from swine kidney tissue, purified it and determined its leucine amidase activity five years later.^3^ The isolation and purification procedures were significantly improved in the late 1960s and early 1970s, among other methods, through the use of sucrose solutions and gel chromatography. The procedure of Pfleiderer^4^ is still used for manufacturing commercially available APN, referred to as leucine aminopeptidase, microsomal.^5^ Pfleiderer also clearly distinguished his purified APN from “classic” leucine aminopeptidase, EC 3.4.11.1, which can also be isolated from swine kidney, exhibits a similar substrate specificity, but is activated by heavy metal ions and belongs to M17 class of peptidases.^4,6^ Its complete amino acid sequence of 967 amino acids was deduced from cloned cDNA in 1988.^1^

### Structure features of APN

The human APN is a protein composed of 967 amino acid residues, with molecular mass of 109 540 Da.^7^ It is known to exist in two forms. The membrane-anchored form, expressed in the renal and intestinal epithelia, the nervous system, myeloid cells, and fibroblast-like cells such as synoviocytes, is commonly referred to as hCD13, while the soluble form present in the blood plasma is known as sCD13.^7,8^ When dividing the hCD13 sequence into three main domains, the topological cytoplasmic domain is located between residues 2 and 8 (sequence: AKGFYIS). The helical transmembrane domain spans amino acids 9 to 32 (KSLGILGILLGVAAVCTIIALSVV). The segment from residues 32 to 66, known as the Ser/Thr-rich stalk, marks the beginning of the extracellular region, or ectodomain, which encompasses the majority of the protein (residues 33–967). The active site is located within this ectodomain.^7^ The hCD13 exists predominantly in a dimeric form, in which two monomers are bound together probably *via* their ectodomains, particularly within their N-terminal parts 636–967.^9^

### Catalytic mechanism and moonlighting

**Fig. 1:**
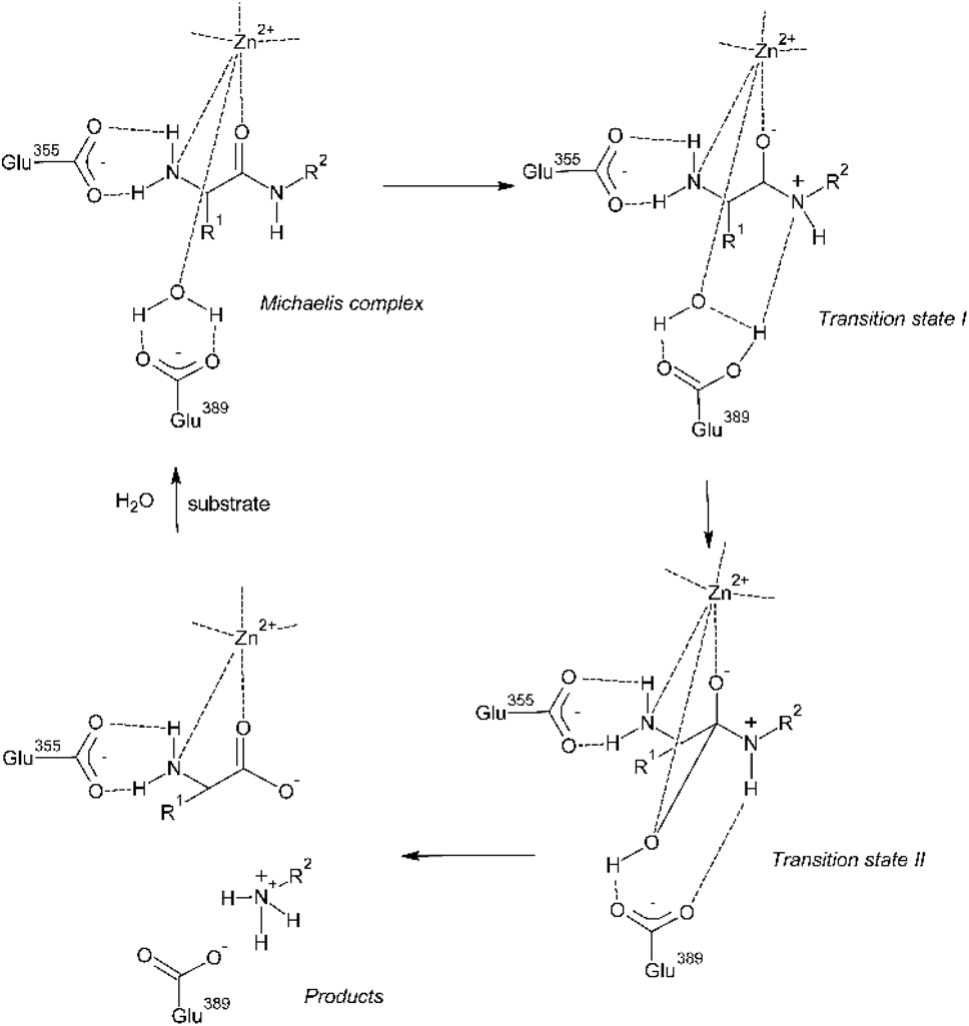
Catalytic mechanism of M1 aminopeptidases based on the activation of water molecule by Zn^2+^ ion. First, H_2_O coordinated to Zn^2+^ is displaced toward Glu389 and the Michaelis complex is formed. The substrate then drives the Zn^2+^-bound H_2_O toward Glu389. In transition state I, the nucleophilicity of H_2_O is enhanced by hydrogen bonds to Glu389 as well as by liganding to Zn^2+^, and it attacks C=O carbon to form pentacoordinate transition state II. H^+^ accepted by Glu389 is then transferred to the leaving nitrogen. The collapse of this intermediate to products is made easier by another H^+^ transfer *via* Glu389. H^+^ is accepted from the hydrated peptide and attached to the leaving nitrogen. Drawn by the authors based on ref. 7.

APN belongs to the M1 family of peptidases which are characterized by the presence of Zn^2+^ ion in their catalytic site. This ion is bound by two histidines and a glutamate. The catalytic mechanism of APN involves activation of a water molecule by the zinc ion (see Fig. 1). The Zn^2+^ ion exhibits an approximately tetrahedral coordination geometry, with three ligands contributed by the protein—His388, His392, and Glu411—and the fourth by a water molecule. The Michaelis complex (see Fig. 1) is formed when the carbonyl oxygen of the scissile bond is positioned near the Zn^2+^ ion. In this complex, both the carbonyl oxygen and the free amino group coordinate with the Zn^2+^, stabilizing the substrate within the active site. The incoming substrate optimizes its interactions in the protein subsites by driving the zinc-bound water molecule toward Glu389 (transition state I in Fig. 1). In this state, both protons of the water molecule form hydrogen bonds with Glu389, enhancing its nucleophilicity, while the oxygen atom remains coordinated to the Zn^2+^ ion. This tripartite interaction orients the remaining lone pair of electrons on the water molecule toward the carbonyl carbon of the substrate, positioning it optimally for nucleophilic attack. The water molecule then attacks the carbonyl carbon, leading to the formation of a pentacoordinate intermediate (transition state II in Fig. 1). The proton accepted by Glu389 is then immediately transferred to the leaving nitrogen. The collapse of this intermediate into the final products is facilitated by a second proton transfer mediated by Glu389. The proton is transferred from the hydrated peptide and shuttled to the leaving nitrogen atom.^7^

APN also performs functions that are independent of its catalytic activity. For example, it acts as a receptor for certain coronaviruses^10,11^ and serves as a receptor for tumor-homing peptides, and it can be a target for inhibiting angiogenesis.^12^ Due to these functions, APN is sometimes classified as a so-called moonlighting enzyme—or more broadly, a moonlighting protein—terms that refer to enzymes or proteins with multiple, distinct functions.^13^

## B. Biological roles and pathophysiological functions

### Physiological substrates

Due to its specificity to N-terminal substrates, APN plays a role in the final biotransformation of peptides generated by hydrolysis of proteins by gastric and pancreatic proteases. APN takes part in metabolism of glutathione (GSH) conjugates. GSH, γ-Glu–Cys–Gly, is a tripeptide utilized by organisms to conjugate both exogenous and endogenous hydrophobic electrophiles, including oxidation products generated during phase I metabolism, facilitating their elimination *via* urine. A compound serving as a conjugation substrate is attached to the sulfur atom of the cysteine residue in GSH through the catalytic action of glutathione S-transferase. Subsequently, the glutamate residue is cleaved from the conjugated tripeptide by γ-glutamyltranspeptidase. The remaining dipeptide is then cleaved by a series of 6 peptidases. APN is one of them,^14^ remaining ones are the “right” leucyl aminopeptidase (pepA, E.C.3.4.11.1), cytosol aminopeptidase (LAP3, E.C. 3.4.11.5), PepB aminopeptidase (E.C. 3.4.11.23), Cys–Gly metallodipeptidase dug1 (E.C. 3.4.13.18) and dipeptidase D (E. C. 3.4.13.20). The resulting Cys-S-conjugate is now N-acetylated by N-acetyltransferase 8 (NAT8, E.C. 2.3.1.80) and final substituted mercapturic acid is excreted as salt with urine.^15,16^ For example, *N-*acetyl-*p-*benzoquinone imine (NAPQI), a toxic minor metabolite of paracetamol (acetaminophen [USAN]), is conjugated with GSH to form 2-(glutathion-*S*-yl)-4-(acetamido)phenol. Glutamate is subsequently removed from the tripeptide by γ-glutamyltranspeptidase, yielding *S*-[5-(acetamido)-2-hydroxyphenyl]cysteinylglycine. This intermediate is then cleaved with the participation of APN to form *S*-[5-(acetamido)-2-hydroxyphenyl]cysteine, which is finally acetylated by NAT8 to produce *S*-[5-(acetamido)-2-hydroxyphenyl]mercapturic acid. The mercapturic acid conjugate is ultimately excreted in urine as its sodium salt.^17^ Inhibition of APN could theoretically impede or slow NAPQI detoxification; however, this function can be compensated by five other peptidases.

APN is reported to be involved in the processing of various peptides, including peptide hormones such as angiotensin III and IV, neuropeptides, and chemokines. Membrane-anchored in various cell types—including neutrophil leukocytes and brain microglial cells—APN participates in the degradation of enkephalins.^18,19^ Neurokinin A (substance K), a decapeptide neuropeptide, has also been identified as a substrate of APN.^20,21^ Neurokinin A and the structurally similar substance P are endogenous ligands of NK-1 receptors, whose antagonists—such as fosaprepitant, aprepitant, and netupitant—are used as antiemetics to manage chemotherapy-induced nausea and vomiting.^22^ Nevertheless, neurokinin A is hydrolyzed by APN, whereas substance P is not,^21^ and works as an APN inhibitor in micromolar concentrations.^23^ Regarding peptides involved in the renin-angiotensin system, angiotensin III (AT III)—a heptapeptide that retains vasoconstrictive and aldosterone-secretion-stimulating activity—is degraded by APN.^24,25^ Angiotensin IV, a hexapeptide which is formed, has been shown to enhance learning and memory by interacting with AT4/IRAP receptors.^25^ Somatostatin, a 116-amino-acid hormone that inhibits the secretion of pituitary hormones—including growth hormone (GH1), prolactin, corticotropin (ACTH), luteinizing hormone (LH), and thyroid-stimulating hormone (TSH)—has also been reported as a substrate of APN.^26^

### Roles in immune system

APN plays a significant role in immune modulation through both its enzymatic activity and receptor functions.^27^

### Role in antigen presentation

APN/CD13 on dendritic cells has been reported to selectively and efficiently degrade exogenously supplied peptide antigens, a process that can be inhibited either by acetylation of the peptide's amino terminus or by bestatin, an APN inhibitor.^29^

### Immune modulation

APN cleaves the N-terminal amino acids of various cytokines and chemokines, thereby modulating their activity and influencing immune responses. This enzymatic function is crucial in regulating the bioavailability and activity of inflammatory mediators. APN/CD13 has been implicated in the activation and differentiation of monocytes and macrophages. It is expressed on the surface of blood monocytes and is upregulated by interferon-γ (IFN-γ), lipopolysaccharides, complement component C5a, interleukin-4 (IL-4), and transforming growth factor-β (TGF-β), while its expression is downregulated by interleukin-10 (IL-10).^30^ APN/CD13 also participates in phagocytic processes in dendritic cells and macrophages. It modulates the phagocytosis mediated by receptors for the Fc portion of IgG antibodies (Fc*γ*Rs).^31^ APN/CD13 is also expressed by fibroblast-like synoviocytes (FLS) present in synovial fluid (SF) in rheumatoid arthritis (RA). Recombinant human CD13 was found chemotactic for cytokine-activated T cells through a G protein-coupled receptor and contributed to the chemotactic properties of SF independently of enzymatic activity. CD13 thus could play an important role as a T cell chemoattractant, in a positive feedback loop that contributes to RA synovitis.^32^ APN has also been identified as a novel negative regulator of antigen-induced mast cell activation, which plays an important role in type I allergy and anaphylactic shock. In a low-dose model of passive systemic anaphylaxis in mice, the antigen-dependent decrease in body temperature—reflecting the severity of the anaphylactic reaction—was significantly exacerbated by the CD13 inhibitor bestatin (−5.9 ± 0.6 °C) and by CD13 deficiency (−8.8 ± 0.6 °C), compared to controls (−1.2 ± 1.97 °C).^33^

### Non-catalytic functions

#### Viral receptor

APN serves as a cell entry receptor for certain coronaviruses. Enveloped coronaviruses engage host receptors *via* their spike (S) glycoprotein, the principal cell entry protein responsible for attachment and membrane fusion.^34^ In humans, alphacoronaviruses that utilize APN as their protein receptor tend to be less pathogenic than certain betacoronaviruses from lineage B—such as SARS-CoV-2, the causative agent of COVID-19—which use angiotensin-converting enzyme II (ACE2) as their entry receptor.^35^

Some other alpha- and deltacoronaviruses that use APN as their receptor endanger animals important to humans, including cattle and pets.^36^ It has been experimentally demonstrated that feline APN (fAPN), which shares 78% sequence identity with human APN and 77% identity with porcine APN, can serve as a receptor not only for feline enteric coronavirus (FeCV) and feline infectious peritonitis virus (FIPV), but also for canine enteric coronavirus (CCV), swine transmissible gastroenteritis virus (TGEV) and human HCV-229 viruses.^37^

In addition to coronaviruses, APN was reported to mediate the infection with human cytomegalovirus (HCMV). Although this virus is distinct from coronaviruses, it is a double-strand DNA virus ordered among *Herpesvirales*, while coronaviruses have only single-strand RNA. The study of Söderberg and colleagues^38^ showed that antibodies targeting human CD13 not only inhibited infection but also blocked binding of HCMV virions to susceptible cells. Furthermore, known APN activity inhibitors such as actinonin, bestatin, 2,2′-dipyridyl and 1,10-phenanthroline, inhibited HCMV infection. The study further demonstrated that various anti-APN antibodies—some targeting the catalytic site and others binding distinct regions—all inhibited APN's capacity to mediate HCMV infection. The authors also prepared a truncated APN lacking 39 amino acids including the catalytic site and expressed it at murine cells (called then hAPNMUT-3T3), and they found that these cells can bind HCMV, but not HCoV-229E. They concluded that distinct binding sites for HCMV and HCoV-229E exist within the APN sequence, and that the catalytic site is not required for the binding of either virus. Interestingly, APN inhibitors that block or bind directly to the catalytic site can also inhibit HCMV binding to APN, regardless of whether the APN contains the complete sequence or lacks the catalytic site.^38^ The role of APN as an HCMV cellular binding receptor was subsequently confirmed in a study demonstrating that HCMV binding to human APN/CD13 inhibits the differentiation of macrophages into monocytes.^39^

#### Angiogenesis in cancer

Angiogenesis or neovascularization, meaning formation of blood vessels or blood vessel-like tubes, is a crucial procedure for cancer growth and development. APN was demonstrated to be overexpressed in tumor neovasculature and to work as target for tumor homing peptides containing NGR (=Asn–Gly–Arg) motif in their sequence. This insight enables the enhancement of anticancer drug activity through its conjugation with an NGR peptide.^12^

## C. APN in disease

APN is highly expressed in various pathology conditions.^1^

### Role in cancer

High levels of APN expression have been observed in a wide range of tumors. APN is among the most extensively studied proteins in the context of oncogenesis and has been specifically linked not only with angiogenic, but also proliferative, metastatic and apoptotic activity of tumors.^13,40^

APN/CD13 has been found to be expressed in non-small cell lung cancer (NSCLC), which accounts for approximately 85% of all lung cancer cases. Two subtypes of NSCLC are recognized: pulmonary adenocarcinoma and squamous cell carcinoma. APN expression was observed in the cell membranes of cancer cells in pulmonary adenocarcinoma, and in the membranes of interstitial cells in squamous cell carcinoma. Positive APN/CD13 expression was detected in 62.3% (43 of 69) of squamous carcinoma cases and in 50% (29 of 58) of adenocarcinoma cases. CD13 expression was found to be significantly correlated with lymph node metastasis and the clinical stage of NSCLC.^41^

Hepatoblastoma is a malignant pediatric tumor originating from immature hepatic cells. APN/CD13 expression was detected in all 30 tissue samples obtained from 16 pediatric patients diagnosed with hepatoblastoma. Prognosis, measured by five-year event-free survival and overall survival, was more favorable in patients with low CD13 expression compared to those with high expression.^42^

The overexpression of APN/CD13 in pancreatic adenocarcinoma (pancreatic cancer, PC) is accompanied with the significant increase of soluble form of APN in blood serum. A clinical study was performed in which 204 PC patients and 178 non-pancreatic cancer subjects including 87 healthy volunteers were engaged. The APN serum concentration in PC patients was significantly higher than in all others.^43^ An increased expression of APN was also immunohistochemically detected in a gallbladder tissue of patients with squamous cell/adenosquamous carcinoma (SC/ASC) together with similarly increased expression of aconitase (E.C. 4.2.1.3), which is also similarly overexpressed. Both enzymes were then proposed as prognostic biomarkers for this rarely identified carcinoma.^44^

In contrast, only marginal APN activity compared to that of dipeptidyl peptidase IV (DPP IV, EC 3.4.13.11) was found in human and porcine follicular thyroid carcinoma cells.^45^

The expression of APN/CDC13 has also been confirmed in other malignancies such as in neuroblastoma, breast ductal carcinoma, and both acute and chronic myeloid leukemia cells.^1^

### Role in inflammation

APN/CD13, along with DPP IV, is expressed in three types of skin cells involved in inflammatory processes, such as those observed in acne vulgaris.

In the SZ95 sebocyte cell line, two DPP-IV inhibitors and the APN inhibitors actinonin and bestatin suppressed proliferation, enhanced terminal differentiation, and slightly decreased total neutral lipid production. The anti-inflammatory and differentiation-restoring cytokine interleukin-1 receptor antagonist (IL-1Ra) was significantly upregulated in SZ95 sebocytes and the HaCaT keratinocyte cell line in the presence of APN and DPP IV inhibitors. Furthermore, the inhibitors suppressed proliferation and interleukin-2 (IL-2) production in *Propionibacterium acnes*-stimulated T-cells *ex vivo*, while enhancing the expression of the immunosuppressive cytokine transforming growth factor-β1 (TGF-β1). These findings provide evidence for a functional role of APN and DPP-IV in the sebaceous gland apparatus and suggest that their inhibitors could affect acne pathogenesis in a therapeutic manner.^46^

In the kidneys, APN is typically anchored in the brush-border membrane of proximal tubule epithelial cells. In cases of acute kidney injury (AKI) APN, along with other peptidases such as glutamyl- (E.C. 3.4.11.7), cysteinyl- (E.C. 3.4.11.3), and asparagyl amino peptidase (E.C. 3.4.11.21), is released to the urine. Assaying its peptidase activity in urine may serve as a useful tool for the early diagnosis of AKI. This was demonstrated in a rat model of AKI induced by cisplatin administration. The assay was performed fluorometrically with alanyl-β-naphthylamide as a fluorogenic substrate.^47^

APN engagement in RA by expression in fibroblast-like synoviocytes (FLS) in synovial fluid was mentioned above under immune modulation.^32^

Systemic sclerosis (SSc) is an autoimmune multisystem disease with a poorly understood etiology. In this context, soluble CD13 (sCD13) is generated through the cleavage of membrane-bound CD13 on monocytes and macrophages by matrix metalloproteinase 14 (MMP14). The resulting sCD13 molecule binds to the bradykinin receptor B1 (B1R), thereby triggering pro-inflammatory, pro-arthritic, and pro-angiogenic responses. This mechanism was elucidated by confirmation of expression of the genes for CD13, B1R, and MMP14, which was elevated in skin biopsies from patients with diffuse cutaneous (dc) SSc. Moreover, single-cell analysis of skin biopsies from SSc patients revealed that BDKRB1 receptor expression is highest in COL8A1-positive myofibroblasts. This type of contractile web-like fusiform cells can be only seen in SSc. TGF-β triggered the expression of BDKRB1 and production of sCD13 by dcSSc skin fibroblasts. Treatment of dcSSc fibroblasts with sCD13 promoted fibrotic gene expression, signaling, cell proliferation, migration, and gel contraction. The pro-fibrotic responses of sCD13 or TGF-β were prevented by a B1R antagonist. In mouse experiments, animals deficient in CD13 or BDKRB1 genes exhibited resistance to bleomycin-induced skin fibrosis and inflammation, a commonly used animal model for SSc. Pharmacological inhibition of B1R produced comparable antifibrotic effects.^48^

### Role in viral infections

Coronaviruses using the APN as their entry receptor such as HCoV-229E circulate in the human population where they are responsible for a significant part of the common cold cases. They use hAPN in epithelial cells of respiratory tract as their cellular receptor.^49^

Pigs can be infected with transmissible gastroenteritis virus (TGEV), porcine respiratory coronavirus (PRCV), porcine epidemic diarrhea virus (PEDV), all of which are caused by alphacoronaviruses. TGEV was decimating for breeding farms with young piglets between 1940s^50^ and 1980s until PRCV appeared in Belgium in 1984. PRCV is a mutant of TGEV that has a 200 amino-acid deletion in the amino-terminus of the S protein. The infection is either asymptomatic, or with mild respiratory symptoms. The deletion in the S protein of PRCV removes the sialic acid binding activity of the virus, preventing PRCV from binding to mucins and mucin-like glycoproteins in the intestine and changing the tissue tropism of the virus to the respiratory tract.^51^ Nevertheless, it provides complete immunity against TGEV, which is why the presence of PRCV in piglet populations has been protecting pig farms from TGEV for several decades.^36^ PEDV belongs to the subgenus *Pedacovirus*. It has been known since 1977.^52^ The disease affects pigs of all ages, although its severity decreases with increasing age. It is very contagious, being transmitted *via* the fecal–oral route or *via* fomites. Mortality rate is between 50% to 100% in newly born piglets.^36^ PDEV infections have caused substantial economic losses in the pork industry in Asia and North America. The last nationwide outbreak in the U.S. in 2013–2014 reduced the pig population by 10%.^53^ Vaccines against PEDV exist. An attenuated oral vaccine available in South Korea and Philippines was made from PEDV DR13 strain and could protect about 50% of pigs.^54^ Porcine deltacoronavirus (PDCoV) causes diarrhea and intestinal lesions in infected piglets but has not traditionally been considered lethal for pigs. Its impact was underestimated until it triggered major diarrhea outbreaks in swine across the United States in 2014. PDCoV can infect cells expressing feline, human, or chicken APN, and young chickens and turkeys have been shown to be experimentally susceptible to infection.^55^ This cross-species transmission is the most concerning feature of this virus, and coronaviruses in general, as it leads to the evolution of new viral strains.^36^

Alphacoronaviruses, which can infect dogs and cats, include canine enteric coronavirus (CCV) and feline enteric coronavirus (FeCV). These two viruses can only cause inapparent or mild infections or mild infections in young animals limited to the enteric tract. Another feline alphacoronavirus, feline infectious peritonitis virus (FIPV), is much more dangerous. It causes a chronic, systemic, and usually fatal disease called feline infectious peritonitis (FIP), characterized by fibrinonecrotic and pyogranulomatous peritonitis and pleuritis. FIPV can also spread to the central nervous system, where it causes granulomatous meningoencephalitis and uveitis.^37^

## D. Diagnostic and theranostic applications

### Plasma sCD13 in cancer diagnosis

Early diagnosis of tumors generally increases the patient's chance of survival and recovery. APN has been reported to be overexpressed in some cancers such as pancreatic cancer (PC)^43^ or hepatoblastoma.^42^ In such cases, blood serum sCD13 concentrations are elevated. A study involving 382 participants with pancreatic cancer (PC) found that serum sCD13 concentration is an independent predictor of patient mortality and overall survival.^43^ Determining plasma sCD13 concentrations is a simple and non-invasive procedure that may aid in early diagnosis; however, it lacks specificity, as elevated levels can also be associated with non-malignant conditions such as systemic sclerosis (SSc).^48^

### Targeting of APN in tumor neovasculature

APN, as a transmembrane surface protein, is highly expressed during tumor-induced angiogenesis, alongside integrins αvβ3 and αvβ5. Peptides containing the RGD (Arg–Gly–Asp) motif, which specifically bind to integrins, and those with the NGR (Asn–Gly–Arg) motif, which selectively target APN, are classified as tumor-homing peptides.^56^ In this context, the primary significance of APN in cancer diagnosis and therapy lies in its role as a target molecule for NGR peptides conjugated with various compounds—either as diagnostic probes for imaging tumor neovasculature or as carriers of antineoplastic agents.

### Fluorescent, PET/SPECT and MRI imaging agents

**Fig. 2:**
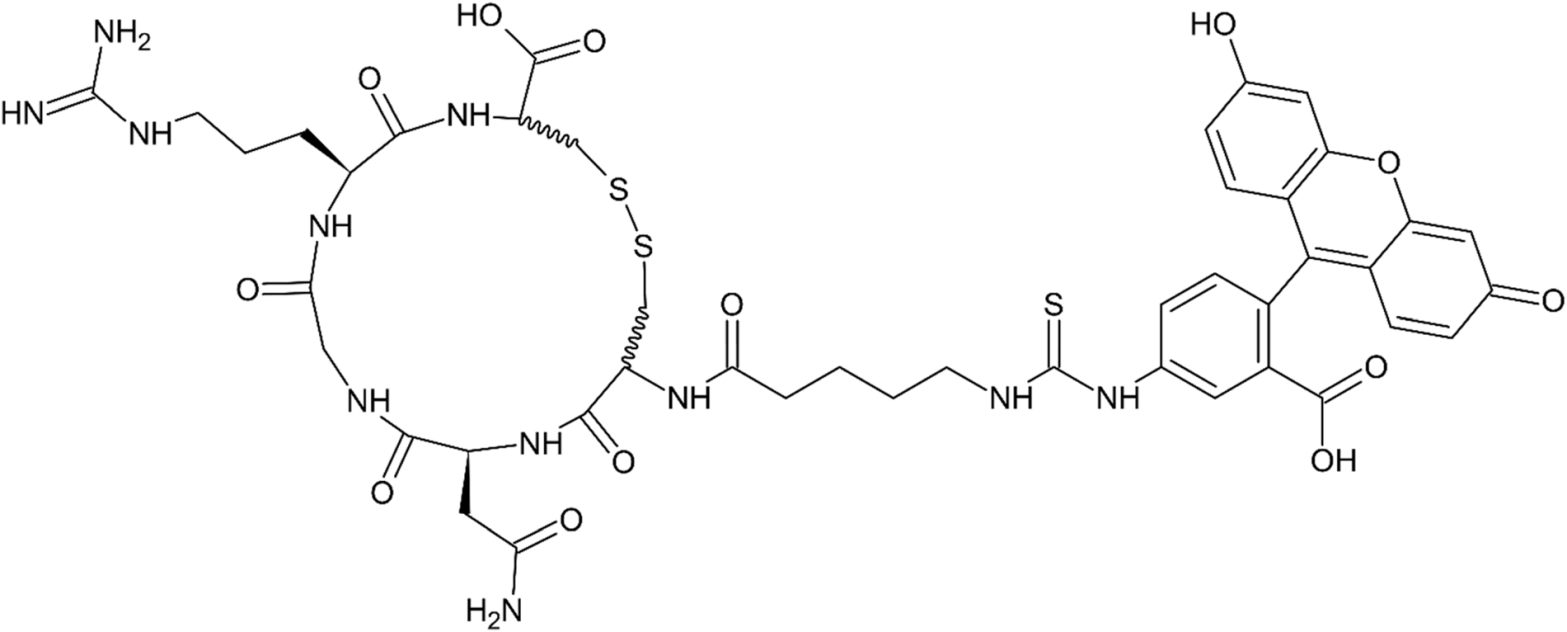
CNP1: a cyclic NGR peptide–fluorescein conjugate using 5-(thioureido)valeroyl linker, an example of probe for fluorescent microscopy imaging of CD13+ tumor neovasculature.^57^

The first reported approach was to synthesize fluorescent probes consisting of a short cyclic peptide containing NGR sequence, attached to fluorescein molecule *via* thiourea containing linker. Three such molecular probes were synthesized and tested for their ability to distinguish between CD13-expressing tumor cells (CD13+: HT-1080 fibrosarcoma cells) and cells without CD13 expression (CD13-: MDA-MB-231, human adenocarcinoma cell line) by means of fluorescent microscopy. One of these conjugates named CNP1 (see Fig. 2) was proposed by authors as a prototype APN/CD13 fluorescent probe for tumor molecular imaging.^57^

**Fig. 3:**
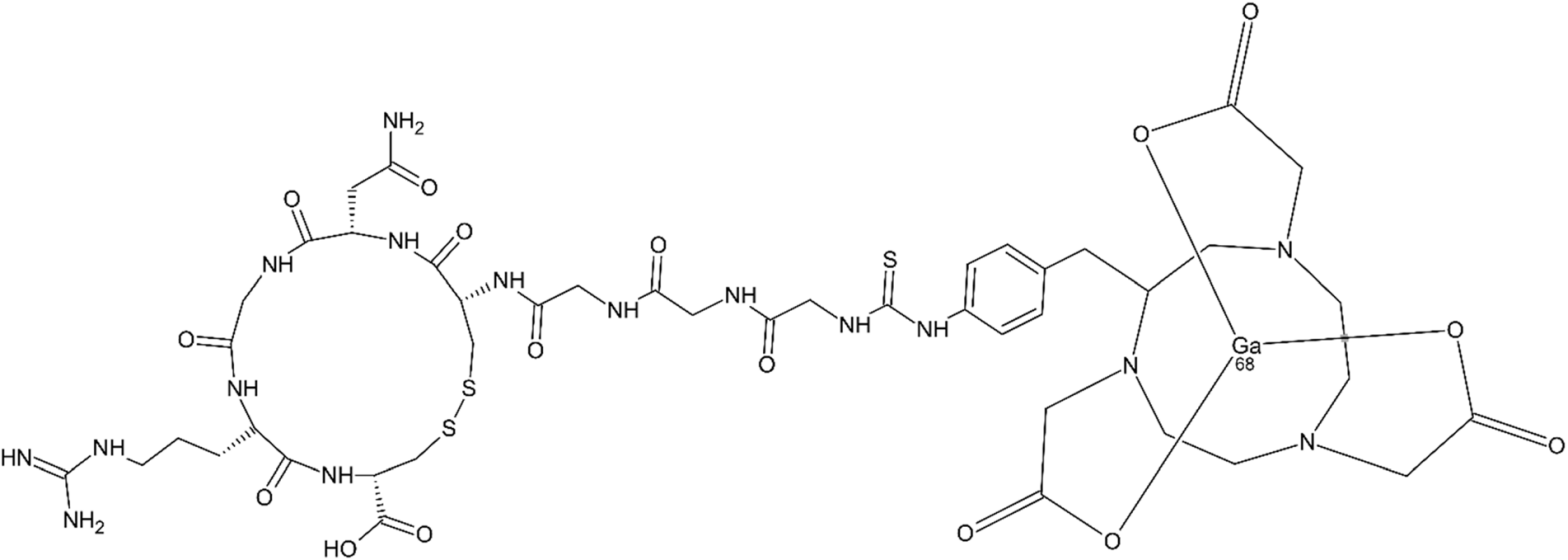
^68^Ga-NOTA-G3-NGR: a cyclic NGR-containing peptide linked with ^68^Ga chelate with NOTA serving as CD13/APN specific probe for PET imaging of a tumor.^59^

NGR peptides labeled with various radionuclides represent a relatively large group of diagnostic probes that have been tested mainly preclinically. For such a purpose, ^68^Ga, ^64^Cu, ^99^Tc, ^188^Re and ^213^Bi have been evaluated to date. Positron emission tomography (PET) and single-photon emission computed tomography (SPECT) were used as imaging methods.^58^ An NGR conjugate of a ^68^Ga chelate with 1,4,7-triazacyclononane-*N*,*N*′,*N*′′-triacetic acid (NOTA), abbreviated as ^68^Ga-NOTA-G3-NGR (Fig. 3), was tested in imaging of CD13+ HT-1080 cells and CD13- HT-29 human colon adenocarcinoma cells for comparison. A nude mouse model was used in combination with PET imaging as the detection method. Significantly higher tumor uptake of the chelate in CD13+ HT-1080 tumors compare to CD13- HT-29 tumors, along with effective blocking in HT-1080 tumors, demonstrating that ^68^Ga-NOTA-G3 -NGR functions as a CD13-specific PET probe.^59^

Another study used ^64^Cu chelated to the “cage” of sarcophagine, *i.e.* 3,6,10,13,16,19-hexaazabicyclo[6.6.6]icosane-1,8-diamine, conjugated *via* linkers to two NGR peptides, for imaging CD13+ HT-1080 cells in comparison to CD13-MCF-7 breast adenocarcinoma cells. The compound was abbreviated as ^64^Cu-Sar-NGR2. This study used the same animal model and the same detection method as the previously mentioned one. The study found that ^64^Cu-Sar-NGR_2_ exhibited strong binding affinity and CD13 specificity in HT-1080 cells, along with excellent tumor uptake in HT-1080 xenografts. Based on these results, the authors concluded that the bivalency effect and optimal molecular size of ^64^Cu-Sar-NGR_2_ make it a promising PET probe and a potential prototype for developing radiometal-based complexes for tumor diagnosis.^60^ An interesting study involving ^99^Tc employed two isomeric hexapeptides containing the NGR sequence—ECG-NGR and NGR-ECG. Notably, the authors did not use an external chelating agent, instead relying on the intrinsic chelating properties of the peptides themselves. The study found that the radiochemical stability of ^99^Tc-NGR-ECG in blood serum was significantly lower than that of ^99^Tc-ECG-NGR at both 5 and 24 hours, whereas their stability in saline was comparable.^61^ This difference in stability may be attributed to enzymatic degradation of the peptide, catalyzed by plasma peptidases such as APN, which is known to hydrolyze the Cys–Gly bond in glutathione precursors.^62^^99^Tc-ECG-NGR demonstrated significant tumor uptake and is therefore considered a promising candidate for tumor imaging.^61^ A metal ion complexed with a chelating agent conjugated to an NGR-containing peptide—targeting CD13/APN expressed in tumor neovasculature can also be utilized for non-invasive tumor diagnosis through magnetic resonance imaging (MRI).

A gadolinium complex, designated CA1, comprises ^68^Gd^3+^ chelated by DOTA [2-(4,7,10-tris(carboxymethyl)-1,4,7,10-tetrazacyclododec-1-yl)acetic acid], which is conjugated *via* an amide bond to the ε-amino group of Lys7 (K7) in the peptide sequence KFDGRGKGGCNGRC. This peptide includes both NGR and RGD sequences, enabling dual targeting of CD13 and α_v_β_3_ integrin—receptors^63^ selectively expressed in tumors and associated with tumor angiogenesis.^64^ The compound was evaluated using A549 human lung adenocarcinoma epithelial cells. The results demonstrated that CA1 exhibits high relaxivity, and that simultaneous targeting of two tumor markers, CD13 and αvβ_3_ integrin, significantly enhances the accumulation of the contrast agent at the tumor surface, thereby improving the quality of imaging. The experimental data indicate that the simultaneous use of RGD and cNGR peptides as tumor-targeting moieties represents a highly promising strategy for the noninvasive clinical diagnosis of tumors.^63^

### Therapeutic conjugates of tumor homing peptides

**Fig. 4:**
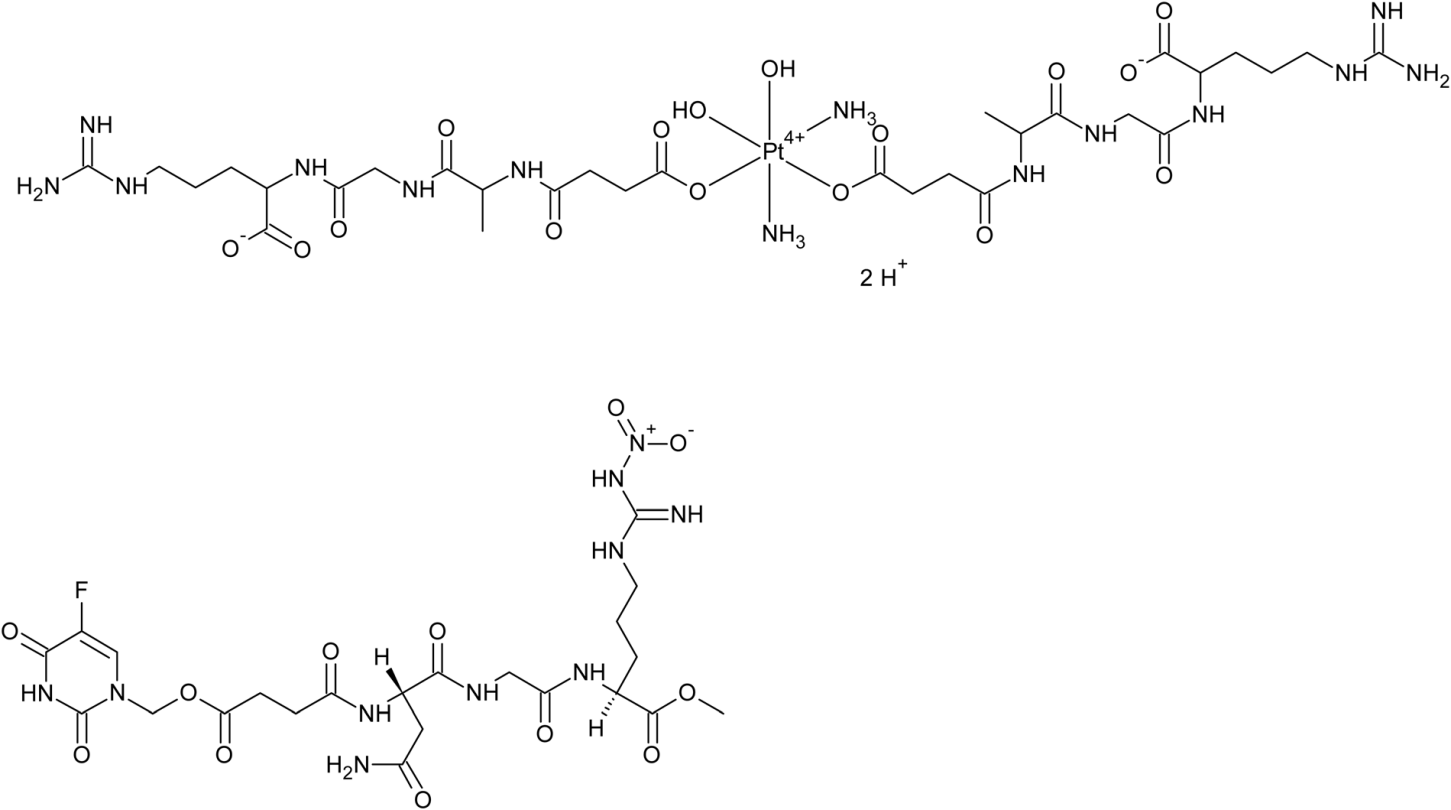
Simple NGR therapeutic conjugates with antineoplastic agents and succinate as a linker: double NGR-conjugate of satraplatin (top)^56^ and NGR-NO_2_ conjugate of 5-fluorouracil (bottom).^66^

NGR double and single conjugates of satraplatin, a Pt(IV) anticancer drug candidate intended for oral administration, which has recently undergone clinical trials,^65^ were more effective at inhibiting the proliferation of two different types of endothelial cells than the analogous satraplatin conjugates with randomly selected tripeptides. However, they were less active than the analogous conjugates with an RGD peptide targeting integrins.^56^ A conjugate of 5-fluorouracil with an NGR peptide (see Fig. 4), bearing a nitro group substituted at the terminal nitrogen of the guanidine moiety of arginine, inhibited angiogenesis in the HCT-116 xenograft female mouse model more effectively than 5-fluorouracil alone.^66^

All above mentioned therapeutic conjugates use succinate as a linker. Subsequently, a series of small cyclic NGR peptide–daunorubicin conjugates with varying stability profiles were synthesized. These conjugates were subsequently evaluated against HT-1080 human fibrosarcoma and HT-29 human colon cancer cell lines. Conjugates in which the drug molecule was attached *via* C-terminal elongation of the cyclic NGR peptides exhibited greater antitumor activity against both cell lines than the free cytostatic agent daunorubicin.^67^

The attempts to conjugate the NGR peptides with anticancer agents were not limited to small molecules. A conjugate with the human tumor necrosis factor α (hTNF-α) has undergone phase II clinical trials for treatment of malignant pleural mesothelioma (MPM). Progression-free survival was monitored as an indicator of therapeutic efficacy; however, the results were inconclusive.^68^

## E. Therapeutic inhibition of APN

### Metabolism of regulatory proteins and AADR

The primary significance of APN as a potential drug target appears to lie in its hydrolytic activity. Cancer cells rely on the efficient turnover of signaling molecules to support their survival, rendering them dependent on specific amino acids (AAs) for proliferation and metastatic potential. APN, along with other members of the M1 family of zinc metalloproteases—such as aminopeptidase A, aminopeptidase B (APB), leukotriene A_4_ hydrolase (LTA4H), and puromycin-sensitive aminopeptidase (PuSA)—contributes to the degradation of proteins^69,70^ including signaling molecules, thereby ensuring the supply of specific amino acids essential for cellular functions. Suppression of this amino acid supply induces deprivation stress in cells, which subsequently activate recovery signaling pathways to mitigate the deficit. This cellular response to amino acid starvation, known as the amino acid deprivation response (AADR), can be triggered by proteasome inhibitors such as bortezomib^70^ or aminopeptidase inhibitors such as tosedostat.^69^ It is characterized by the upregulation of AA synthase genes, AA transporters and tRNA synthetases. Despite these interventions, however, AA deficiency persists, leading to both caspase-dependent and caspase-independent apoptosis, which is the intended outcome of anticancer therapy. Nevertheless, the effect of AADR on the tumor microenvironment is still poorly understood;^71^ however, this cannot deter medicinal chemists from designing molecules that induce AADR as potential anticancer agents.

### Inhibitor classes

An overview of APN inhibitors, which have undergone various levels of testing for therapeutically relevant activities, ordered in accordance with their chemical structure.

### Peptides and pseudopeptides

**Fig. 5:**
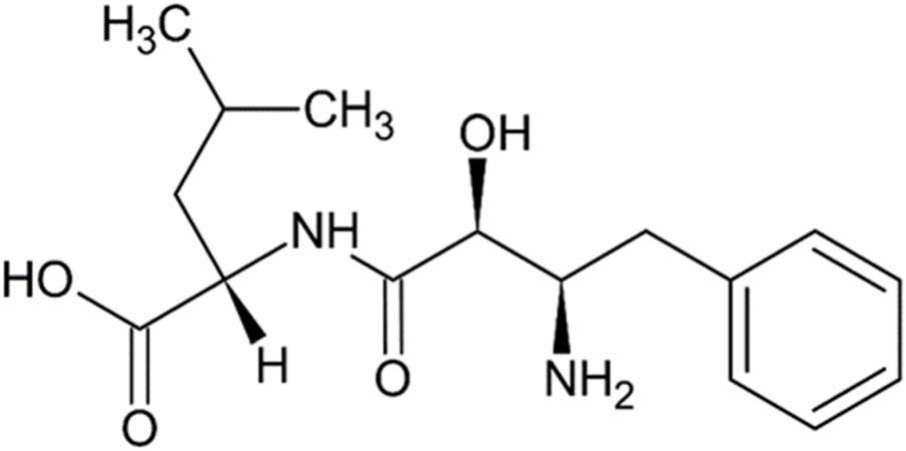
Bestatin/ubenimex, a dipeptide isolated from *Streptomyces olivoreticuli*, the most known APN inhibitor, tested and used for various therapeutic activities.^72–83^

Bestatin or ubenimex [INN], (2*S*)-2-[[(2*S*,3*R*)-3-amino-2-hydroxy-4-phenylbutanoyl]amino]-4-methylpentanoic acid (Fig. 5), was first isolated from a culture filtrate of a Gram-positive bacteria *Streptomyces olivoreticuli* from the class of *Actinomycetes*^72^ and its chemical structure has been subsequently elucidated.^73^ It significantly inhibits APN, but also APB, LTA4H,^74,75^ PuSA^76^ (all involved in AADR triggering^70^ and other peptidases. Bestatin has been undergoing numerous clinical trials, from phase I to phase III, since the 1980s and continues to be studied today. It was, for example, effective against different types of lung^77,78^ or gastric^79^ cancer or myelodysplastic syndrome and chronic leukemia.^80^ Recently, it was tested for non-cancer issues such as pulmonary arterial hypertension, lower limb lymphedema or ischemic stroke.^81^ Bestatin is officially listed in the Japanese Pharmacopeia, which includes two articles devoted to it: one on the substance itself and another on bestatin-containing capsules.^82^ It was approved and marketed in Japan as an immunomodulatory and antitumor drug under the trademark Ubenimex (Nippon Kayaku Co., Ltd., Tokyo), around 2001,^83^ and designated as an orphan drug for pulmonary arterial hypertension in EU in 2016.^75^ Regarding the mechanism of bestatin binding to the active site of APN, Glu350 seems to be important as it binds its protonated NH_3_^+^ with its free γ-carboxyl,^27^ while OH and CONH groups are chelated to Zn^2+^ by their O atoms, which is held at the active site chelated to His383, His387 and Glu406 residues. Benzene ring of the benzyl substituent is proposed to dwell in the hydrophobic pocket between Glu350 and His383, and the isobutyl chain of the Leu part of bestatin is in another pocket nearby Glu406.^27^

**Fig. 6:**
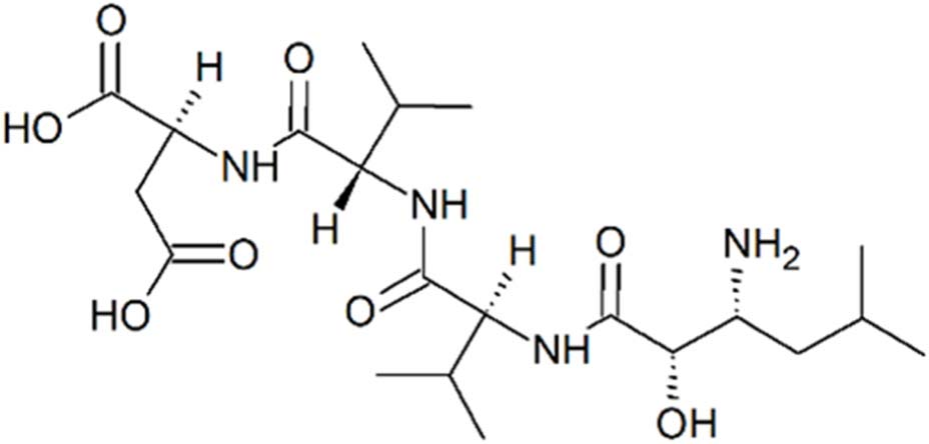
Amastatin, a tetrapeptide aminopeptidase inhibitor from *Streptomyces* ME 98-M3.^84–89^

Amastatin (Fig. 6) is a tetrapeptide, in particular (2*S*,3*R*)-3-amino-2-hydroxy-5-methylhexanoyl-l-Val–l-Val–l-Asp.^84^ It also comes from *Streptomyces* genus, strain code ME 98-M3.^85^ In addition to APN, it also inhibits APB and LAP. It has been reported to potentiate the behavioral effects of the peptide hormones oxytocin and vasopressin, likely by inhibiting their degradation.^86^ It has been used in some *in vitro* studies aimed at elucidating the regulatory roles of aminopeptidases in the body.^87,88^ Its mechanism of APN inhibition is presumed to be similar to that of bestatin.^89^

**Fig. 7:**
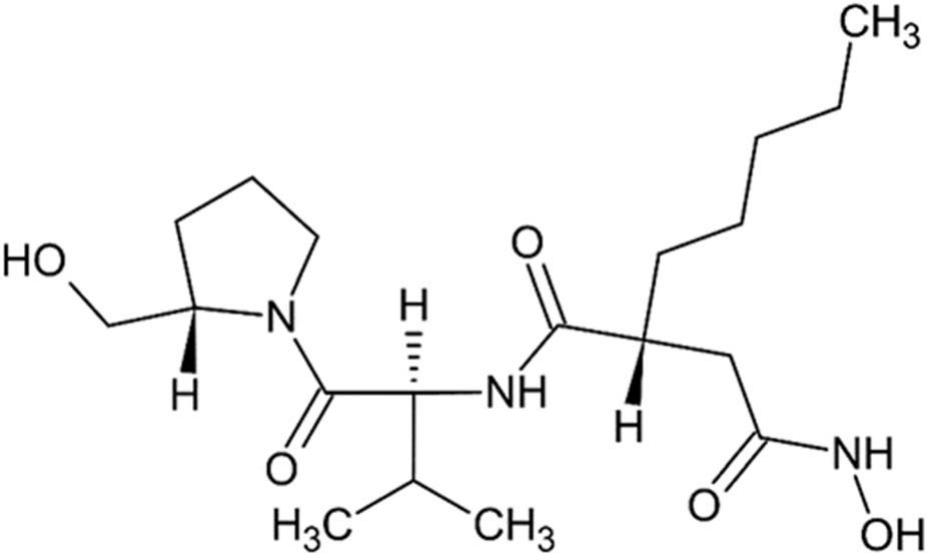
Actinonin, a pseudotripeptide hydroxamate inhibitor of Zn-hydrolases from *Streptomyces roseopallidus*.^89^

Actinonin, a pseudotripeptide (Fig. 7), (2*R*)-*N*′-hydroxy-*N*-[(2*S*)-1-[(2*S*)-2-(hydroxymethyl)pyrrolidin-1-yl]-3-methyl-1-oxobutan-2-yl]-2-pentylbutanediamide (Fig. 7), is a product of *Streptomyces roseopallidus*. It is a rare example of a naturally occurring compound with hydroxamic moiety.^89^ In addition to APN, it also inhibits peptide deformylase (PDF; EC 3.5.1.88), a metallo-hydrolase that contains a Zn^2+^ ion in its active site and is regarded as a promising target for antibacterial therapy. Actinonin interacts with PDF active site *via* its hydroxamate head group.^90^ It is likely that it inhibits APN through a similar mechanism.

**Fig. 8:**
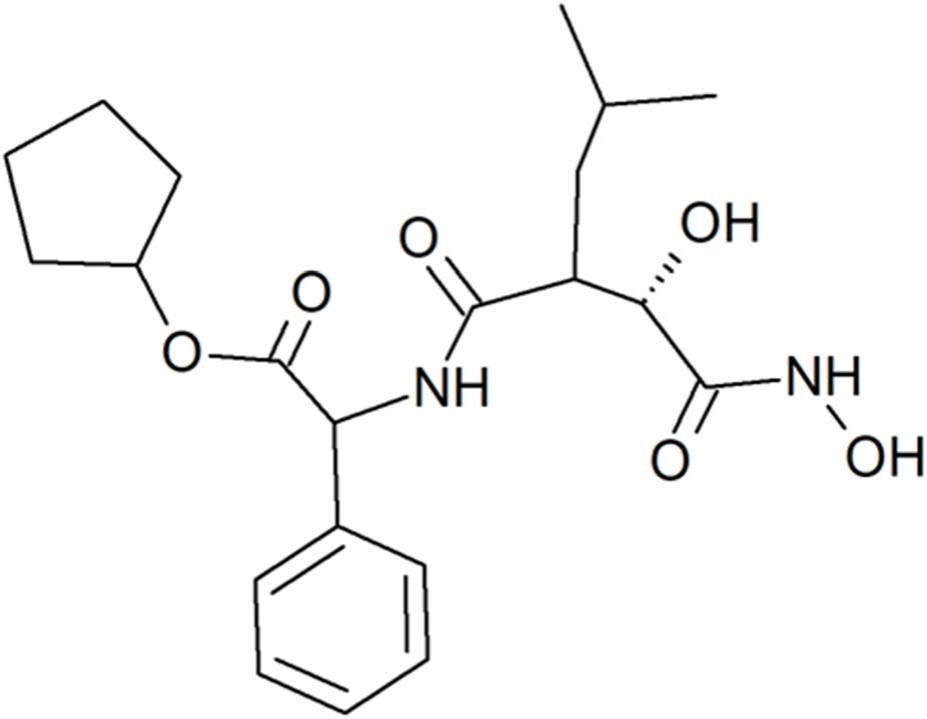
Tosedostat, a cyclohexyl ester prodrug of a hydroxamate dipeptide, which passed clinical trials for liquid and solid malignancies.^69,91,92^

Tosedostat or CHR-2797, cyclopentyl (2*S*)-2-[[(2*R*)-2-[(1*S*)-1-hydroxy-2-(hydroxyamino)-2-oxoethyl]-4-methylpentanoyl]amino]-2-phenylacetate (Fig. 8), is a synthetic dipeptide also with a hydroxamate group. It is a cyclopentyl ester prodrug of CHR-79888, (2*S*)-({(2*R*)-2-[(1*S*)-1-hydroxy-2-(hydroxyamino)-2-oxoethyl]-4-methylpentanoyl}amino)(phenyl)acetic acid, to which it decomposes after entering a cell.^69^ It inhibits several M1 class aminopeptidases such as APN, PuSA and LTA4 hydrolase. It may be considered an analogue of actinonin, exhibiting improved oral bioavailability. Tosedostat has been evaluated in clinical trials for the treatment and supportive care of acute myeloid leukemia (AML) and other forms of leukemia, as well as multiple myeloma, non-small cell lung cancer, pancreatic cancer, and other malignancies, either as monotherapy or in combination with other anticancer drugs.^91,92^

### Fused ring system compounds

#### Nitrogenous heterocycles

**Fig. 9:**
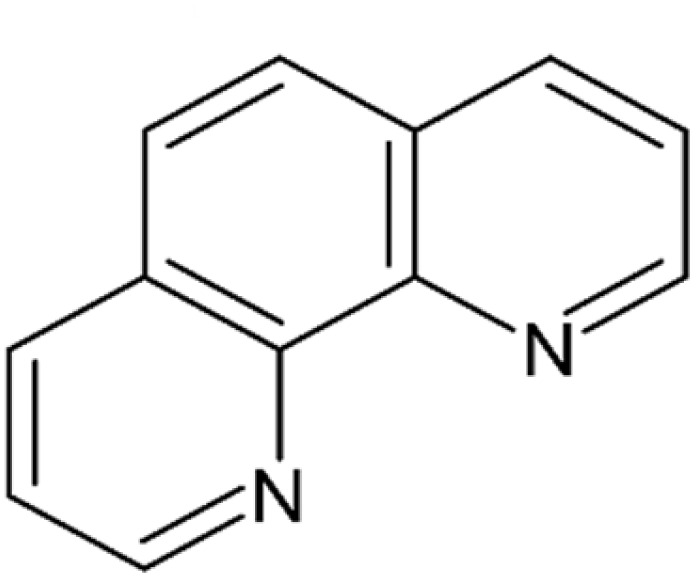
1,10-Phenanthroline – a representative of polycyclic nitrogenous heterocycles inhibiting zinc metallopeptidases.^38,94^

The group of isomeric phenanthrolines, *i.e.*, diazaphenanthrenes, is here represented by 1,10-phenanthroline or 4,5-diazaphenanthrene (Fig. 9). It is a synthetic compound obtained *via* Skraup synthesis, utilizing *o*-phenylenediamine and glycerol.^93^ It inhibits several zinc-dependent metallopeptidases, including APN^38^ and plasmodial aminopeptidase Ey (APEy; EC 3.4.11.20—also referred to as aminopeptidase N, which can cause terminological confusion^94^) as well as other members of the M1 peptidase family,^38^ and pyroglutamyl peptidase (PGP), an enzyme involved in the degradation of thyrotropin-releasing hormone (TRH).^95^ The compound is a well-known potent chelator of divalent and trivalent metal cations,^96^ with its inhibitory mechanism involving coordination to the Zn^2+^ ion within the enzyme's active site. While 1,10-phenanthroline is useful as a standard for laboratory assays, it lacks sufficient selectivity and potency to be considered a viable drug candidate.

#### Cyclic triterpenoids

**Fig. 10:**
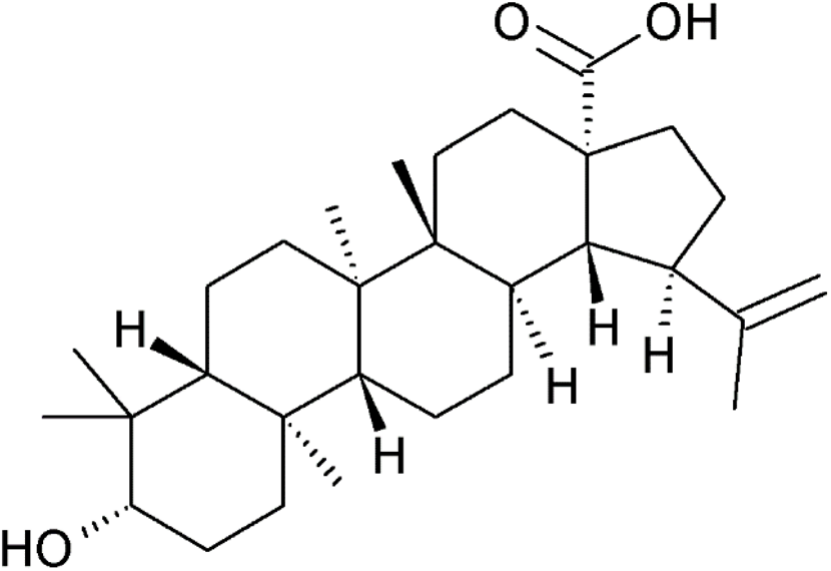
Betulinic acid, a triterpenoid secondary metabolite of birches, an APN inhibitor in a dose-dependent manner.^97–101^

Betulinic acid, 3-hydroxylup-20(29)-en-28-oic acid (Fig. 10), is a pentacyclic triterpenoid found in several species of birch (*Betula*) and other plants. However, it is often prepared semi-synthetically from betulin (lup-20(29)-ene-3β,28-diol) through careful oxidation with chromium(vi) oxide, followed by reduction of the keto group at position 3 using sodium tetrahydroborate.^97^ The bark of common European birch species, such as *Betula pendula,* contains up to 35% of betulin, which can be extracted from it.^98^ Betulinic acid has been reported to inhibit APN activity in a dose-dependent manner. This inhibitory activity is higher than that of bestatin (IC_50_ 7.3 μM *vs.* 16.9 μM). Its inhibitory activity against aminopeptidases appears to be selective and restricted to APN. The compound has undergone clinical evaluation for the treatment of dysplastic nevus syndrome, a condition regarded as a precursor to melanoma. The compound was applied as a 20% ointment.^99^ Betulinic acid has demonstrated various types of *in vitro* anticancer activity. Its capacity to induce apoptosis in cancer cells has been attributed to multiple mechanisms, including the inhibition of aminopeptidase N (APN).^100^ Recently, the compound has been proposed as a therapeutic agent for glioblastoma, delivered topically *via* formulations such as nanoparticles or ionic liquids.^101^

#### Amino benzosuberones

**Fig. 11:**
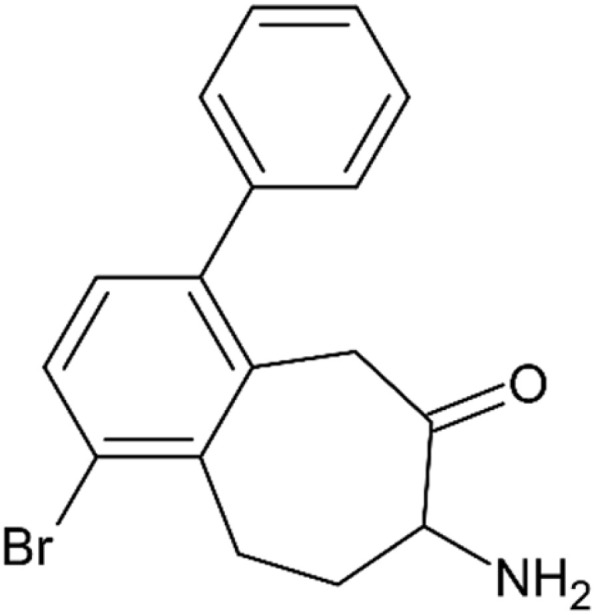
7-Amino-1-bromo-4-phenyl-5,7,8,9-tetrahydro-6*H*-benzo[7]annulen-6-one (CHEMBL1852660), the compound with the highest inhibition activity against APN from all reported inhibitors.^102^

7-Amino-1-bromo-4-phenyl-5,7,8,9-tetrahydro-6*H*-benzo [7]annulen-6-one, or 7-amino-1-bromo-4-phenylbenzosuberone (Fig. 11) is probably the most potent reported APN inhibitor at all. This compound has been given an ID CHEMBL1852660 of the European Bioinformatics Institute EMBL-EBI Data Base.^102^ It was the most potent inhibitor within a series of structurally related compounds, which included 1,4-disubstituted 7-amino-5,7,8,9-tetrahydro-6*H*-benzo[7]annulen-6-ones, 9-amino-7,8,9,11-tetrahydro-10*H*-cyclohepta[*a*]naphthalen-10-one, and its isomer, 9-amino-7,9,10,11-tetrahydro-8*H*-cyclohepta[*a*]naphthalen-8-one. *K*_i_ of CHEMBL1852660 against bovine kidney APN reached 60 pM.^103^ It also exhibited excellent selectivity in the inhibition of APN as a representative of one-zinc aminopeptidase. Its *K*_i_ values for two-zinc leucine aminopeptidase (LAP, E.C. 3.4.11.1) and aminopeptidase from *Aeromonas proteolytica* (E.C. 3.4.11.10) were 70 μM and 39 μM respectively.^103^ The APN inhibitory activity of CHEMBL1852660 was later assessed on porcine APN, yielding results similar to those for bovine APN (*K*_i_ = 60 pM). However, its inhibitory activity was less potent against human and mouse APN, with *K*_i_ values of 350 pM and 200 pM, respectively.^104^ Nevertheless, these values are still considered very good.

At a concentration of 1 μM, CHEMBL1852660 significantly inhibited capillary tube formation in the HUVEC angiogenesis model, outperforming bestatin at 300 μM as well as two analogues from the 7-amino-5,7,8,9-tetrahydro-6*H*-benzo[7]annulen-6-one series, which ranked just below CHEMBL1852660 in APN inhibitory activity.^104^

### Schiff bases

#### Oximes

**Fig. 12:**
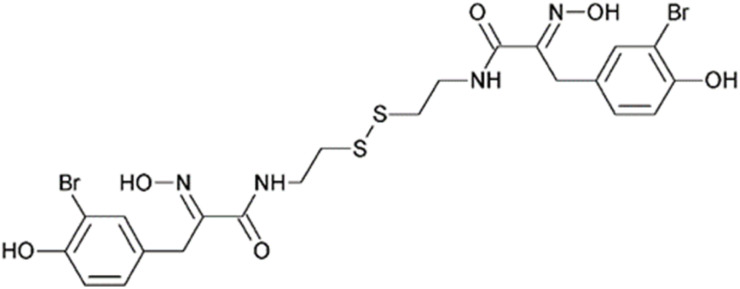
Psammaplin A, a disulfide dioxime from marine sponges with wide activity against metalloenzymes.^27,98,105,106^

Psammaplin A, (2*E*)-3-(3-bromo-4-hydroxyphenyl)-*N*-[2-[2-[[(2*E*)-3-(3-bromo-4-hydroxyphenyl)-2-hydroxyiminopropanoyl]amino]ethyldisulfanyl]ethyl]-2-hydroxyiminopropanamide (Fig. 12), is a natural peptidases inhibitor isolated from marine sponges *Psammaplysilla* sp. and *Dysidea* sp. It is a rare example of an APN inhibitor of animal origin.^27^ Its two oxime groups exhibit strong binding affinity for the Zn^2+^ ion.^98^ In addition to APN, it also inhibits sortase A and B (EC 3.4.22.70 and EC 3.4.22.72), transpeptidases responsible for anchoring surface proteins to the bacterial cell wall, which are considered promising targets for antibacterial therapy.^105^ Here, the binding of the oxime groups is likely directed toward coordination with the Ca^2+^ ion.

It was found to inhibit porcine microsomal APN activity in a non-competitive manner, with an IC_50_ value of 18 μM. It also demonstrated inhibitory effects on the proliferation of several distinct cancer cell lines. This effect was significantly more pronounced in cell lines with high APN expression. Additionally, it inhibited bFGF-induced angiogenesis in BAECs cultured on Matrigel, in a dose-dependent manner.^106^

#### Semicarbazones and thiosemicarbazones

**Fig. 13:**
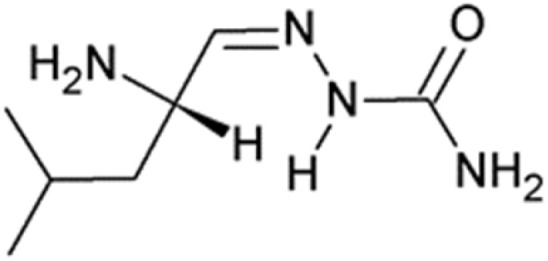
l-Leucinal semicarbazone, a patented activator of regulatory T-cells.^107^

l-Leucinal semicarbazone, or (2*E*)-2-[(2*S*)-2-amino-4-methylpentylidene]hydrazinecarboxamide (Fig. 12), with a reported *K*_i_ of 230 mM against APN, has been patented as an activator of regulatory T cells (Treg cells; CD4+CD25+) for the treatment of autoimmune disorders such as type 1 diabetes and multiple sclerosis. The therapeutic strategy involves upregulating TGF-β1 expression in and on Treg cells—an effect that, according to the patent,^107^ can be induced by APN inhibition through L-leucinal semicarbazone and other known APN inhibitors, including bestatin, amastatin, and actinonin (Fig. 13).^107^

**Fig. 14:**
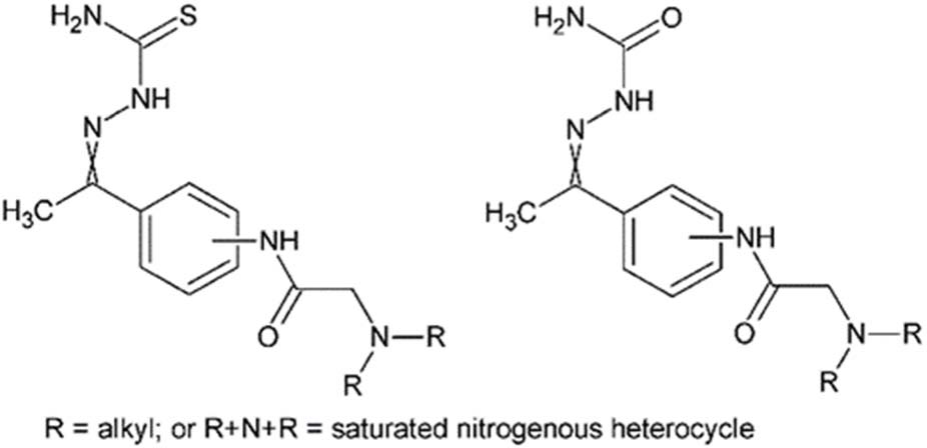
Semicarbazones and thiosemicarbazones derived from basically substituted aminoacetophenones inhibiting APN and cancer cell lines proliferation.^108^

A series of semicarbazones and thiosemicarbazones, derived from basically substituted acetamidophenones, was recently synthesized as potential APN inhibitors. The compounds differed in the nature of the terminal tertiary amino group, which was either a dialkylamino moiety or a saturated nitrogen-containing heterocycle, as well as in the position (*ortho*, *meta*, or *para*) of the substituted acetamido group relative to the *N*-substituted 1-iminoethyl moiety. The latter moiety was either a semicarbazone or a thiosemicarbazone, depending on the specific compound (Fig. 14).^108^

The compounds were initially tested for their inhibitory activity against porcine kidney APN, and the most effective ones were subsequently evaluated for antiproliferative activity against cancer cell lines, both APN-positive and APN-negative, using WST-1 assay. Compounds, 4-[2-(4-benzylpiperazine-1-yl)acetamido]acetophenone thiosemicarbazone and 24-5-3, 4-[2-(pyrrolidine-1-yl)acetamido]acetophenone thiosemicarbazone exhibited APN inhibitory activity with IC_50_ values of 22.3 μM and 23.5 μM, respectively. Their antiproliferative activity was observed against THP-1 monocytic leukemia cells expressing APN, with IC_50_ values of 66.05 μM and 70.18 μM; against MCF-7 breast carcinoma cells, also expressing APN, with IC_50_ values of 39.94 μM and 75.47 μM, respectively; but showed minimal activity against DU-145 prostate cancer cells lacking APN expression, with IC_50_ values > 100 μM for both compounds. Overall, thiosemicarbazones exhibited higher activity than their corresponding semicarbazones within the series. The proposed mechanism of APN inhibition is the same as in the previously mentioned cases – chelation of Zn^2+^ cation.^108^ The ability of thiosemicarbazone and semicarbazone groups to chelate Zn^2+^ has been repeatedly reported in the literature.^109,113^

### Summary of APN inhibitors

A summary of the key APN inhibitors discussed, categorized by chemical class and accompanied by their inhibitory activities and selectivity profiles, is presented in Table 1.

**Table 1:** Summary of APN inhibitors mentioned in the text with their structure codes, activity, selectivity, and references for activity (first) and selectivity. Unless otherwise stated, the inhibition activity against pig kidney APN expressed as IC_50_ is reported^b^

Name/code	Structure class	Structure as SMILES	Figure no.	APN inhibition IC_50_/*K*_i_ [μM]	Selectivity: other targeted hydrolases	References
Bestatin/ubenimex [INN]	Peptides	CC(C)C[C@@H](C(=O)O)NC(=O)[C@H]([C@@H](CC1=CC=CC=C1)N)O	5	16	APB, PuSA, **LTA4H**	27 and 72–83
Amastatin	Peptides	CC(C)C[C@H]([C@@H](C(=O)N[C@@H](C(C)C)C(=O)N[C@@H](C(C)C)C(=O)N[C@@H](CC(=O)O)C(=O)O)O)N	6	0.5	APB, LAP	27 and 84–86
Actinonin	Peptides	CCCCC[C@H](CC(=O)NO)C(=O)N[C@@H](C(C)C)C(=O)N1CCC[C@H]1CO	7	2.0	PDF	89, 90 and 114
Tosedostat	Peptides/hydroxamates	CC(C)C[C@H]([C@@H](C(=O)NO)O)C(=O)N[C@@H](C1=CC=CC=C1)C(=O)OC2CCCC2	8	0.22^a^	PuSA, LTA4H	91, 92 and 115
1,10-Phenanthroline	Fused ring system compounds/phenanthrolines	C1=CC2=C(C3=C(C=CC=N3)C=C2)N=C1	9	430.2	APEy, PGP	38, 95 and 116
Betulinic acid	Fused ring system compounds/cyclic tritepenoids	CC(=C)[C@@H]1CC[C@]2([C@H]1[C@H]3CC[C@@H]4[C@]5(CC[C@@H](C([C@@H]5CC[C@]4([C@@]3(CC2)C)C)(C)C)O)C)C(=O)O	10	7.3	(None, selective)	98 and 100
CHEMBL1852660	Fused ring system compounds/amino benzosuberones	C1CC2=C(C=CC(=C2CC(=O)C1N)C3=CC=CC=C3)Br	11	6 × 10^−7^^a^	LAP, E.C. 3.4.11.10	103
Psammaplin A	Oximes	C1=CC(=C(C=C1C/C(=N\O)/C(=O)NCCSSCCNC(=O)/C(=N/O)/CC2=CC(=C(C=C2)O)Br)Br)O	12	18	Sortase A and B	105 and 106
l-Leucinal semicarbazone	Semicarbazones	NC(=O)N/N=C/[C@@H](N)CC(C)C	13	230	(None reported)	107
24-10-3	Thiosemicarbazones	O=C(CN1CCN(CC1)Cc2ccccc2)Nc3ccc(cc3)C(/C)=N\NC(N)=S	14	22.3	(None reported)	108
24-5-3	Thiosemicarbazones	S=C(Nc1ccc(cc1)C(/C)=N\NC(N)=S)CN2CCCC2	14	23.5	(None reported)	108

a IC_50_ for a human recombinant APN.

b APB – aminopeptidase B, E.C.3.4.11.6; PuSA – puromycin-sensitive aminopeptidase, E.C. 3.4.11.14; LTA4H – leukotriene A4 hydrolase, E.C. 3.3.2.6, LAP – leucine aminopeptidase, E.C.3.4.11.1; APEy – aminopeptidase Ey, E.C. 3.4.11.20; PGP – pyroglutamyl peptidase, E.C. 3.4.19.3; sortase A, E.C. 3.4.22.70; sortase B, E.C. 3.4.22.71.

### The principals of inhibition and general SAR in APN inhibitors

Zn^2+^ cation chelation is the only mechanism of APN inhibition repeatedly mentioned in the literature. In peptides containing an α-hydroxy-β-amino acid, such as bestatin or amastatin, the Zn^2+^ cation is reported to coordinate with both the hydroxyl group and the carbonyl oxygen of the amide bond. Additionally, it remains coordinated to His383, His387, and Glu406 residues within the APN active site.^27^

In tosedostat or its metabolite CHR-79888 (tosedostat-acid), the isosteric hydroxamate group functions as a metal-chelating moiety.^110–112^ In this case, the Zn^2+^ cation is likely coordinated by the carbonyl and hydroxyl oxygen atoms, similar to its interaction with homologous plasmodial peptidases such as PfA-M1 or APEy.^117^ The inhibitory activity increases by approximately one order of magnitude compared to that of bestatin and amastatin.

Replacement of this group with two pyridine nitrogen atoms separated by two aromatic carbon atoms, as in 1,10-phenanthroline, results in a decrease in activity by three orders of magnitude. 1,10-Phenanthroline has been reported to completely chelate and remove the Zn^2+^ ion from the enzyme.^118^

The aldehyde-semicarbazone moiety, as exemplified by L-leucinal semicarbazone, confers approximately the same level of inhibitory activity.^107^ An improvement in activity by approximately one order of magnitude compared to l-Leucinal semicarbazone can be achieved by substituting the aldehyde-semicarbazone group with an oxime moiety, as seen in psammaplin A,^106^ or with a ketone-thiosemicarbazone, as exemplified by thiosemicarbazones derived from base-substituted acetophenones.^108^

The difference in activity between thiosemicarbazones and semicarbazones—both containing isosteric nitrogen-based chelating groups—can be attributed to the facile formation of a covalent S–Zn bond in the thiol tautomer of thiosemicarbazones.^109,113^

**Fig. 15:**
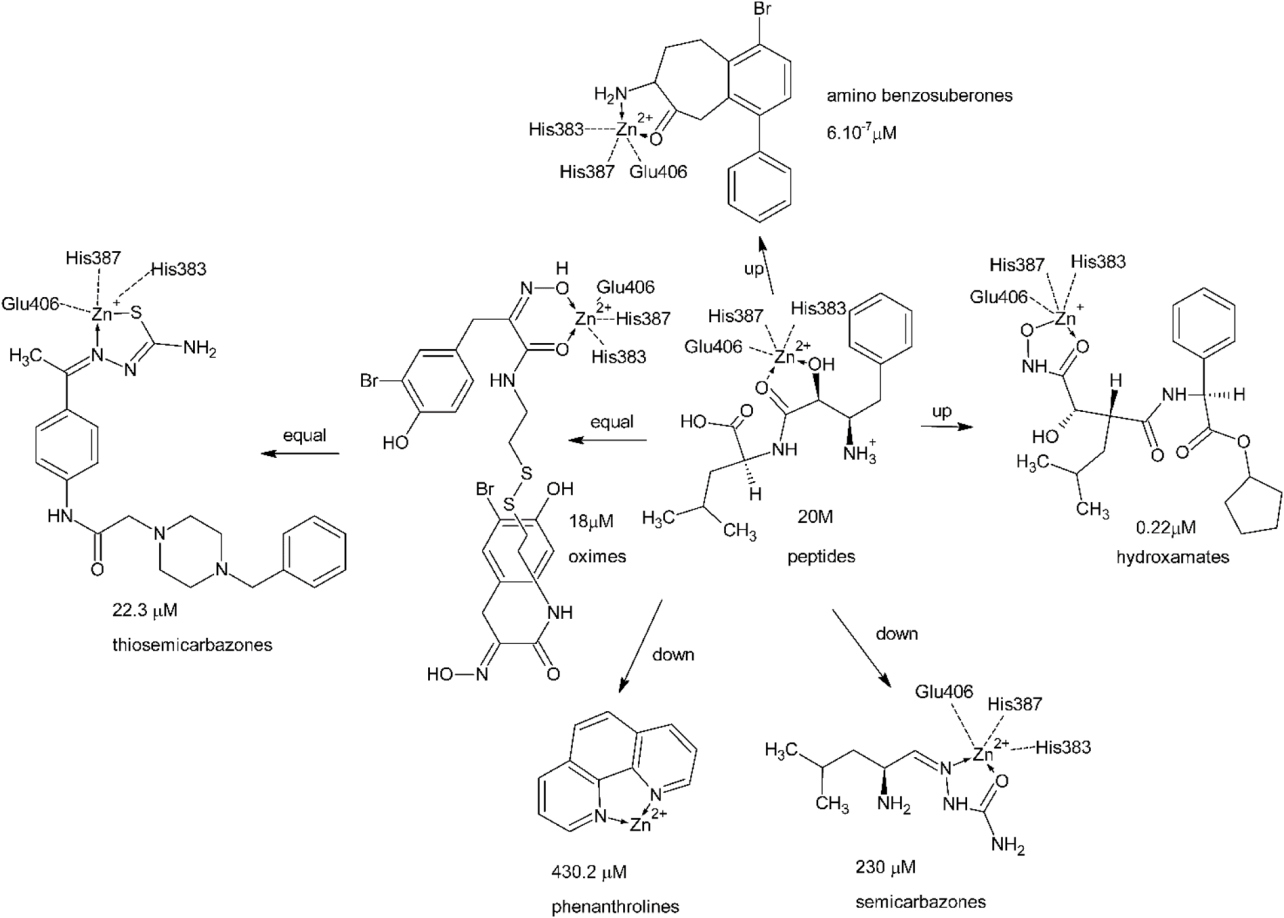
Structure–activity relationships (SAR) across the structural groups of APN inhibitors with Zn^2+^ chelation as the mechanism of activity (arranged by the authors using mainly^27,103,106,109,113,118^).

Replacement of the α-hydroxy carbonyl moiety in the initial peptide inhibitors with an isosteric α-aminoketone group, positioned on the cycloheptane ring fused to the benzene core, results in an increase in activity of six orders of magnitude. Compounds such as CHEMBL1852660 exhibit the highest inhibitory activity overall.^103^ A detailed explanation of this enhanced activity necessitates molecular modeling analyses grounded in precise X-ray crystallography data (see Fig. 15).

Substituents on the inhibitor scaffold capable of forming ion pairs with complementary charged groups of the protein, such as carboxylate and amino residues, can enhance inhibitor binding. Additionally, functional groups that act as hydrogen bond donors or acceptors, as well as lipophilic moieties fitting into hydrophobic pockets of the enzyme, contribute to improved affinity and specificity.^27^ Betulinic acid does not conform to the conventional Zn^2+^ chelation mechanism, suggesting that its inhibitory activity may involve a distinct mode of action.

### Selectivity, dual-action compounds, off-target effects and strategies to improve activity of APN inhibitors

Most APN inhibitors lack selectivity; owing to their metal-chelation mechanism, they often inhibit other M1-class aminopeptidases as well as various other metalloenzymes (compare Table 1). This lack of selectivity may, in fact, represent an advantage by increasing the likelihood of the compound's successful translation into clinical practice.

This is exemplified by the two most therapeutically successful inhibitors, bestatin and tosedostat. They both inhibit LTA4H in addition to APN. LTA4H exhibits dual hydrolase activity: it preferentially cleaves tripeptides at arginyl bonds and catalyzes the hydrolysis of the epoxide ring in leukotriene A4, producing leukotriene B4 with a hydroxyl group at the C-5 position.^119^ LTA4H inhibition is identified as the sole mechanism of action in the EMA Public Summary of Opinion on the orphan designation of bestatin for the treatment of pulmonary arterial hypertension in the EU. Nevertheless, the document mentions that, as of the request for this opinion in 2016, bestatin (ubenimex) was authorized for cancer treatment in Japan, Korea, and China.^75^ In these countries, bestatin is indicated as a maintenance-strengthening therapy following complete remission induction in adult patients with acute non-lymphocytic leukemia, aimed at prolonging survival.^120^ The anticancer activity of bestatin is primarily attributed to its ability to induce amino acid deprivation response (AADR) through inhibition of APN and the peptidase activity of LTA4H; however, inhibition of the epoxide hydrolase function of LTA4H may specifically contribute to its therapeutic effect in colorectal cancer.^74^ Notably, bestatin has been reported to exacerbate the severity of methemoglobinemia induced by anilide and ester local anesthetics—including articaine, benzocaine, bupivacaine, butacaine, butamben, chloroprocaine, cinchocaine, and cocaine—when co-administered. This effect can be attributed to bestatin's inhibition of hydrolases responsible for the hydrolysis of these ester and amide local anesthetics.

This compound may also increase the risk and severity of bleeding when co-administered with acenocoumarol.^121^ This side effect is unlikely to be related to inhibition of lactone ring hydrolysis, as such a metabolic pathway is not known for acenocoumarol or other coumarin derivatives. Acenocoumarol is primarily metabolized *via* hydroxylation at positions 6, 7, and 8 of the coumarin ring by CYP2C9 and CYP2C19 enzymes.^122^

The immunomodulatory activity of bestatin has not been fully elucidated and is therefore considered an off-target effect.^123^ Bestatin exerts effects on cellular immunity, including shifts in T-cell subpopulations that favor helper T cells (CD4^+^) and cytotoxic T cells (CD8^+^), while suppressing regulatory T cells (Tregs),^124^ as well as inhibition of T-cell proliferation in response to mitogenic stimulation. Additionally, bestatin activates monocytes and macrophages, promotes the release of anti-inflammatory cytokines, and suppresses the production of pro-inflammatory cytokines and chemokines—including interleukin (IL)-6, CXCL8/IL-8, and CCL3/macrophage inflammatory protein (MIP)-1α—in lipopolysaccharide (LPS)-stimulated monocytes.^123^ The beneficial immunomodulatory effects of bestatin have been repeatedly demonstrated. Bestatin has also been successfully evaluated as an immunoadjuvant in an inactivated whole-virus vaccine against foot-and-mouth disease (FMD), a highly contagious condition affecting cloven-hoofed animals.^125^

Recently, bestatin was shown to alleviate high-altitude-induced cerebral edema (HACE) in mice by protecting the integrity of the blood–brain barrier (BBB).^126^

Bestatin also shows potential as a therapeutic agent in dentistry for the treatment of periodontitis, as *in vitro* studies have demonstrated its selective bacteriostatic activity against *Porphyromonas gingivalis*—a key pathogen in periodontitis—by inhibiting its growth and biofilm formation without affecting commensal bacteria.^127^

The limited adoption of bestatin as an anticancer agent appears to be due more to its insufficient efficacy than to concerns regarding adverse effects. The anticancer efficacy of bestatin may be enhanced through the design of prodrugs with improved tissue permeability or increased selectivity for cancerous tissues. LYP, the dimethylaminoethyl ester of bestatin, demonstrated greater inhibition of ovarian carcinoma cell growth and more effective suppression of APN activity compared to bestatin itself.^128^ LYP inhibited the growth of ES-2 cells, which express high levels of APN/CD13, significantly more than that of SKOV-3 cells, which lack APN/CD13 expression. This suggests that LYP suppresses cell growth primarily through APN inhibition.^128^

Tosedostat itself is already a cyclopentylester prodrug of CHR-79888, which is (2*S*)-({(2*R*)-2-[(1*S*)-1-hydroxy-2-(hydroxyamino)-2-oxoethyl]-4-methylpentanoyl}amino)(phenyl)acetic acid. Tosedostat exhibits antiproliferative effects against a broad range of tumor cell lines both *in vitro* and *in vivo*, with potency at least 300-fold greater than that of the prototypical aminopeptidase inhibitor, bestatin.^115^ Tosedostat enters cells *via* passive diffusion and is retained intracellularly following esterase-mediated conversion to its hydrophilic active metabolite, CHR-79888. This metabolite induces amino acid depletion and triggers the amino acid deprivation response (AADR). Carboxylesterases (CES), particularly CES1, function as prodrug-activating enzymes, with CES1 expression observed in acute myeloid leukemia (AML) specimens. Two novel myeloid leukemia sublines, U937/CHR2797(200 μM) and U937/CHR2797(5 μM), exhibiting low (14-fold) and high (270-fold) levels of resistance to CHR2797, respectively, have been identified. The highly drug-resistant subline exhibited a complete loss of CES-mediated prodrug activation, correlated with downregulation of CES mRNA and protein expression, significant intracellular retention and sequestration of the prodrug, a marked increase in intracellular lipid droplets, and predominant activation of the pro-survival Akt/mTOR signaling pathway. These findings elucidate the molecular basis of CHR2863 resistance and suggest novel strategies to overcome this drug resistance in myeloid leukemia cells.^129^

In general, strategies to mitigate unwanted off-target effects of APN inhibitors include the development of prodrugs with enhanced organ or tissue selectivity, the design of analogs with improved enzyme inhibition activity, and the conjugation of inhibitors to specific antibodies for targeted delivery.

An alternative approach to potentiate APN inhibitory activity and overcome resistance in malignancies involves combination therapy, such as co-administration of tosedostat with statins—including simvastatin, fluvastatin, lovastatin, and pravastatin—for the treatment of aminopeptidase-resistant acute myeloid leukemia (AML).^130^ The therapeutic efficacy of APN inhibitors in cancer treatment can be enhanced through synergy with other anticancer agents, such as paclitaxel. Combination therapy with paclitaxel and tosedostat has demonstrated improved efficacy against solid tumors; however, this approach is also associated with an increased incidence of adverse effects.^131^ A phase II study evaluating the combination of tosedostat with either cytarabine or decitabine for prolonging survival in older patients with untreated acute myeloid leukemia (AML) or high-risk myelodysplastic syndrome (MDS) yielded inconclusive results.^132^

### Pharmacokinetics of bestatin

Bestatin is well absorbed orally. Following a single 30 mg oral dose administered to healthy male subjects, the peak serum concentration reaches approximately 2.2 μg mL^−1^ at 1 hour post-dose. The drug is nearly completely eliminated from the body within 24 hours. Urinary excretion of unchanged bestatin accounts for 67–73% of the administered dose over 24 hours. Identified metabolites include (2*S*,3*R*)-3-amino-2-hydroxy-4-phenylbutyric acid, representing 9–25%, and *p*-hydroxy-bestatin, comprising 2–5% of the excreted compounds.^120^

### Pharmacokonetics of tosedostat

A dose-proportional increase in plasma AUC and *C*_max_ for both tosedostat and its active metabolite CHR-79888 has been observed over a daily dose range of 10 to 320 mg. The half-life of tosedostat ranges between 1 and 3.5 hours, while that of its active metabolite CHR-79888 ranges from 6 to 11 hours. CHR-79888 accumulates in blood cells at intracellular concentrations consistent with preclinical efficacy. Tosedostat exhibits a high binding affinity to blood plasma proteins.^133^ When co-administered with paclitaxel, the overall exposure (AUC) to tosedostat and CHR-79888 remained largely unchanged, although slight alterations in *C*_max_, *T*_max_, and half-life were observed.^131^ The specific route of elimination of the compound from the body has not been explicitly reported in any study.

## F. Discussion, conclusions and perspectives of APN ligands as drugs

APN can serve as a tumor diagnostic marker either by quantifying its plasma concentration or, more effectively, as a molecular target for conjugates comprising NGR peptides linked to fluorescent, radiolabeled, or MRI-detectable probes. Such conjugates enable selective imaging of tumor neovasculature. APN inhibitors, whether isolated from bacterial or plant sources or synthesized chemically, demonstrate significant therapeutic potential. Several have advanced through preclinical and clinical evaluations and are currently used or designated as orphan drugs for rare diseases—for example, bestatin for pulmonary arterial hypertension^75^ or tosedostat for acute myeloid leukemia.^134^

All reported APN ligands to date with potential as drug candidates act as inhibitors. Their known or potential *in vitro* antiproliferative activity is likely linked to AADR *via* enzyme inhibition.^104,108^ Previously published reviews^27,98,135^ listed a plethora of inhibitors of various structure types. Among these, several representative molecular classes were selected as potential lead compounds for the development of therapeutic agents.

We did not attempt to select the most potent reported molecules due to variability in activity assessment methods and units (*e.g.*, IC_50_*versus K*_i_, mM *versus* mg mL^−1^),^27^ which complicates objective comparison of their inhibitory effects. Furthermore, inhibitory activity can vary significantly between APN enzymes extracted from different species, even when their sequences differ only marginally. This is clearly illustrated by CHEMBL1852660, whose inhibitory activity against human APN is six-fold lower than against bovine APN.^103,104^

The previous reviews also ignore the aspect of chemical stability. Aldehydes and thiols, which are frequent in these reviews about APN inhibitors,^27,98^ lack sufficient stability to be processed into viable formulation or stored for a reasonable duration. Moreover, the aldehyde group can be classified as a toxicophore.^136^ We did not rank these compounds in our selection of inhibitors.

Another critical property is adequate water solubility, which is essential primarily for laboratory assays—for example, measuring APN inhibitory activity by monitoring the decrease in absorbance of 4-nitroaniline produced during the enzymatic hydrolysis of l-leucine-*p*-nitroanilide, a chromogenic substrate. The insufficient effector solubility can be solved by addition of a solubilizer, such as dimethyl sulfoxide or *N-*methylpyrrolidine-2-on. However, limited solubility can complicate both the assay procedure and the accuracy of data interpretation. To address this, chromatographic techniques such as HPLC may be employed as an alternative to conventional visible spectrophotometry.^108^

Our subsequent selection criterion was the anticipated or demonstrated oral bioavailability of the compounds. In this context, strict adherence to Lipinski's rules^137^ was not enforced, as the inhibitors discussed herein are considered primarily as lead structures rather than fully optimized drug candidates. For example, betulinic acid is presented here although it reaches log *P* = 6.64 (ref. 138) which is a bit higher than Lipinski upper limit. However, it can be structurally modified to optimize its lipophilicity.

Finally, we took into account the compounds' inhibitory activities against other peptidases, alongside their potential therapeutic effects on additional relevant biological targets. On the other hand, we did not include compounds with multiple non-specific weak activities such as flavonoids.^27^

Regarding the mechanism of inhibition, the only one frequently cited in the literature is Zn^2+^ cation chelation. Functional groups—including amino groups adjacent to carbonyls, oximes, semicarbazones, thiosemicarbazones, and hydroxamic acids—have been reported to participate in this chelation.^27,90,98,103,139^ This complexation mechanism appears to be applicable to virtually all inhibitors, with the possible exception of betulinic acid.

The aforementioned suggests that inhibitors APN retain their relevance as potential therapeutic agents, particularly for cancerous diseases. Moreover, the initial screening for porcine microsomal APN inhibition is relatively straightforward and does not necessitate a specialized biochemistry laboratory. The measurement of inhibitory activity, *via* hydrolysis of l-leucine-*p-*nitroanilide by an effector and observed as a decrease in absorbance of the resulting 4-nitroaniline at 405 nm,^89,103,108^ is feasible in any medicinal chemistry laboratory equipped with at least a VIS spectrophotometer. The enzyme is commercially available and relatively inexpensivesive.^5^ The structures and SAR presented here, along with the results of molecular docking based on the APN–ligand complexes, which are available,^28^ may serve as a source of inspiration for medicinal chemists to design and modify new molecules that interact with APN. In the design of novel potential anticancer agents, it is generally undesirable to restrict the inhibition spectrum exclusively to APN, as concurrent inhibition of other M1 aminopeptidases may confer therapeutic advantages. Conversely, extending the inhibitory profile to other zinc-dependent enzymes implicated in carcinogenesis, such as histone deacetylase (E.C. 3.5.1.98)^140^ and carbonic anhydrase IX (E.C. 4.2.1.1).^141^ However, enhancing the inhibitory activity specifically against APN is essential.

APN as a moonlighting enzyme has several functions, some of which are unrelated to its enzymatic activity.^13^ It functions as a cellular receptor facilitating the entry of certain coronaviruses and human cytomegalovirus (HCMV). Theoretically, APN ligands developed as potential antiviral agents against certain coronaviruses—primarily those infecting animals—and human cytomegalovirus (HCMV) need not inhibit the enzymatic activity of APN. Nevertheless, such drug candidates appear marginal, as animal coronavirus outbreaks are typically addressed by culling entire livestock populations, and individual severe feline infections with FIPV by euthanasia. Conversely, HCMV represents a promising target for APN ligands as therapeutics. Currently available antiviral agents—such as ganciclovir, its prodrug valganciclovir, cidofovir, and foscarnet—function primarily as viral polymerase inhibitors. However, these agents are associated with significant adverse effects, including neutropenia and nephrotoxicity, as well as a considerable risk of drug interactions,^142^ but no anti-cytomegalovirus drugs targeting APN are currently under development. The sole aminopeptidase studied in relation to HCMV is endoplasmic reticulum aminopeptidase 1 (ERAP1, E.C. 3.4.1.B10), a zinc metallopeptidase belonging to the M17 family. ERAP1 processes HCMV-derived peptides to optimize their presentation to CD8+ cytotoxic T lymphocytes, and the virus produces multiple microRNAs that downregulate ERAP1 expression in host cells, thereby facilitating immune evasion by impairing antigen processing and presentation. Therefore, inhibition of ERAP1 is undesirable; instead, strategies aimed at restoring or enhancing its function may be more beneficial in counteracting HCMV immune evasion.^143^ This presents a significant opportunity for medicinal chemists: to design APN ligands that effectively block its role as a viral receptor without inhibiting the enzymatic activity of ERAP1.

It is generally easier to detect a molecule's inhibitory activity against the APN enzyme than to confirm its binding to the APN protein in the absence of enzymatic inhibition. However, established techniques exist to address this challenge. For example, NMR methods such as differential line broadening and transferred NOE (nuclear Overhauser effect) have been employed for over two decades to characterize ligand–protein interactions without relying on enzyme activity assays.^144^

Targeting APN continues to present a valuable opportunity for medicinal chemists in the development of novel and effective therapeutic agents.

## Author contributions

O. Farsa reviewed the literature and compiled the draft of the manuscript. T. Uher drew the diagrams and optimized spelling, syntax and style of the text.

## Conflicts of interest

There are no conflicts of interest to declare.

## Data Availability

No primary research results, software or code have been included and no new data were generated or analyzed as part of this review.
